# Development of the Rheumatoid Arthritis Distress Scale (RADS): a new tool to identify disease-specific distress in patients with Rheumatoid Arthritis

**DOI:** 10.1186/s41927-021-00220-4

**Published:** 2021-11-16

**Authors:** Linda Silke, Othman Kirresh, Jackie Sturt, Heidi Lempp

**Affiliations:** 1 Outpatient Physiotherapy Department, Mile End Hospital, Bancroft Rd, London, E1 4DG UK; 2 Chelsea and Westminster Hospital, 369 Fulham Road, London, SW10 9NH UK; 3 Florence Nightingale Faculty of Nursing, Midwifery and Palliative Care, King’s College London, James Clerk Maxwell Building, Waterloo, London, SE1 8WA UK; 4 Department of Inflammation Biology, Faculty of Life Sciences and Medicine, Centre for Rheumatic Diseases, Weston Education Centre, King’s College London, 10, Cutcombe Road, London, SE5 9RJ UK

**Keywords:** Distress, Patient Reported Outcome Measure, Qualitative research, Rheumatoid Arthritis, Secondary analysis

## Abstract

**Background:**

Patients with Rheumatoid Arthritis (RA) may experience psychological distress (depression, anxiety) in addition to their physical symptoms. People with RA may also experience disease-specific distress (DSD), related to the specific burden of living with their life-long condition. DSD is a patient reported outcome in several long-term conditions, including type 1 and 2 diabetes. The aims of this study were to determine whether DSD is experienced by people with RA, and if so, develop a Patient Reported Outcome Measure (PROM) to assess for DSD in people with RA.

**Methods:**

A five-phased qualitative study was conducted which consisted of a secondary data analysis of 61 interviews of people with rheumatological disease (Phase 1), validation of findings via a Patient and Public Involvement (PPI) group of people with RA (n = 4) (Phase 2), item generation for a PROM (Phase 3) and establishing face and content validity of the PROM via PPI group (n = 4) and individual cognitive interviews (n = 9) of people with RA respectively (Phase 4 and 5). The final PROM was presented at a Patient Education Evening for patients with long-term rheumatological conditions, including RA, and carers.

**Results:**

Five themes of rheumatological disease distress emerged from Phase 1, which were validated in the Phase 2 PPI group. After Phases 3–5, the Rheumatoid Arthritis Distress Scale (RADS) was formed of 39 items and 3 supplementary questions. Overall participants reported the content of the RADS to be clear and relevant, and that DSD is a valid concept in RA, distinct from other entities like clinical depression or anxiety.

**Conclusions:**

DSD appears to be an important concept in RA. The 39-item RADS demonstrates acceptable face and content validity in this patient group. Further psychometric testing is needed. The RADS may be a useful tool for healthcare professionals to identify RA distress.

## Background

Rheumatoid Arthritis (RA) is a progressive inflammatory disease, which causes pain, joint damage and disability, and affects 0.5–1% of the adult population [1]. Patients with RA commonly experience psychological distress in addition to their physical symptoms, and increased prevalence of depression and anxiety has been reported in RA populations [2–4].

Disease-specific distress (DSD) is one form of psychological distress, which has been identified in several different long-term conditions such as cancer [5–7], type 1 and 2 diabetes [8], and Inflammatory Bowel Disease (IBD) [9]. DSD refers to the distress or burden of living with a particular long-term condition, its symptoms and/or treatments. People with DSD exhibit signs of subjective stress that are not necessarily consistent with those of a diagnosable mental health condition [10]. This means that patients can have DSD without being diagnosed with depression or anxiety [6, 9, 11].

In diabetes, where the evidence is most established, higher levels of DSD, not depressive symptoms or clinical depression, are associated with out of target blood glucose levels [12], and interventions that effectively reduce diabetes distress can improve patients’ glycaemic control [13, 14]. Diabetes distress is an expected part of living with a complex long-term condition. It is not psycho-pathologised nor viewed as diabetes comorbidity [15]. It can be effectively addressed as part of routine disease-specific clinical care by diabetes focused practitioners [13, 15].

DSD in long-term rheumatological conditions has not yet been described in the literature. Given the progress in the detection and management of diabetes distress, and the positive clinical outcomes following effective interventions [12, 13], this current study is important for patients who experience distress when diagnosed with RA, and for their clinicians to offer evidence-based based treatment. The development of a Patient Reported Outcome Measure (PROM) to identify DSD in people with RA seems therefore timely as a first step towards comprehensive care and management.

The aims of this study were to 1. determine if there is any evidence of DSD in patients with RA and 2. if so, to develop a PROM to identify DSD in people with RA. The specific objectives delivered in five phases were to:

1. Identify the presence, or otherwise, of DSD in four existing qualitative rheumatological diseases datasets.
2. Validate findings with a Patient and Public Involvement (PPI) group of patients living with RA.
3. Use the evidence from phases 1 and 2 to generate items for a PROM assessing RA distress.
4. Assess the face and content validity and redundancy with a PPI group of patients with RA.
5. Establish face and content validity of the PROM with scale naive participants.

## Methods

### Study design

This was a five-phased qualitative research study, which aimed to follow previous research methods in developing PROM’s [16–20] and use criteria for reporting qualitative research [21].

In Phase 1, a secondary thematic qualitative data analysis was undertaken retrospectively from 61 audio-recorded 1:1 interview transcripts from four existing data sets, with interview studies dating 2004–2015 [22–25]. Primary research questions focused on the impact of fatigue and inactivity in Idiopathic Inflammatory Myositis (IIM) and Antiphospholipid Syndrome (APS), and the experiences, expectations and needs of patients with RA about their disease management [22–25]. In Phase 2, a PPI group of people with RA (n = 4) was set up to explore and validate the findings of the thematic analysis. In Phase 3, items for a PROM were generated from the identified themes of rheumatological disease distress. In Phase 4, a PPI group of people with RA (n = 3) was consulted with the aim of establishing face and content validity of the measure and performing initial item reduction. In Phase 5, the PROM was presented to people with RA and individual cognitive interviews (n = 9) were conducted to further establish face and content validity, refine items where necessary and ensure the PROM ‘made sense’ to participants. The final draft was presented at a Patient Education Evening for patients with long-term rheumatological conditions, including RA, and carers.

### Ethical considerations

All four anonymized original data sets used in Phases 1–3 had received written ethics approval [22–25]. Author H.L. was an investigator in the primary research for all four studies. Following review of the original protocols it was determined that the aims of the secondary analysis were closely aligned with the original studies’ overall aims for which written consent was previously obtained. Original participants were not therefore approached to re-consent. Transcripts from the original data sets were previously de-identified prior to the original analysis and remained that way for the further analysis. This current research was conducted prior to the stated disposal date for data collected between 2004 and 2015 [22–25].

The local University Ethics Committee provided ethics approval for Phases 3–5 on 24.04.2018 (REC Number: MRS-17/18-6443). Participants undertook written informed consent procedures. Cognitive interviews were transcribed, following removal of identifiable data, by L.S. and a professional transcription service. All audio files were deleted following transcription. All face-to-face interviews and PPI groups were held in a private room in a Medical School.

### Study sample

**Fig. 1:**
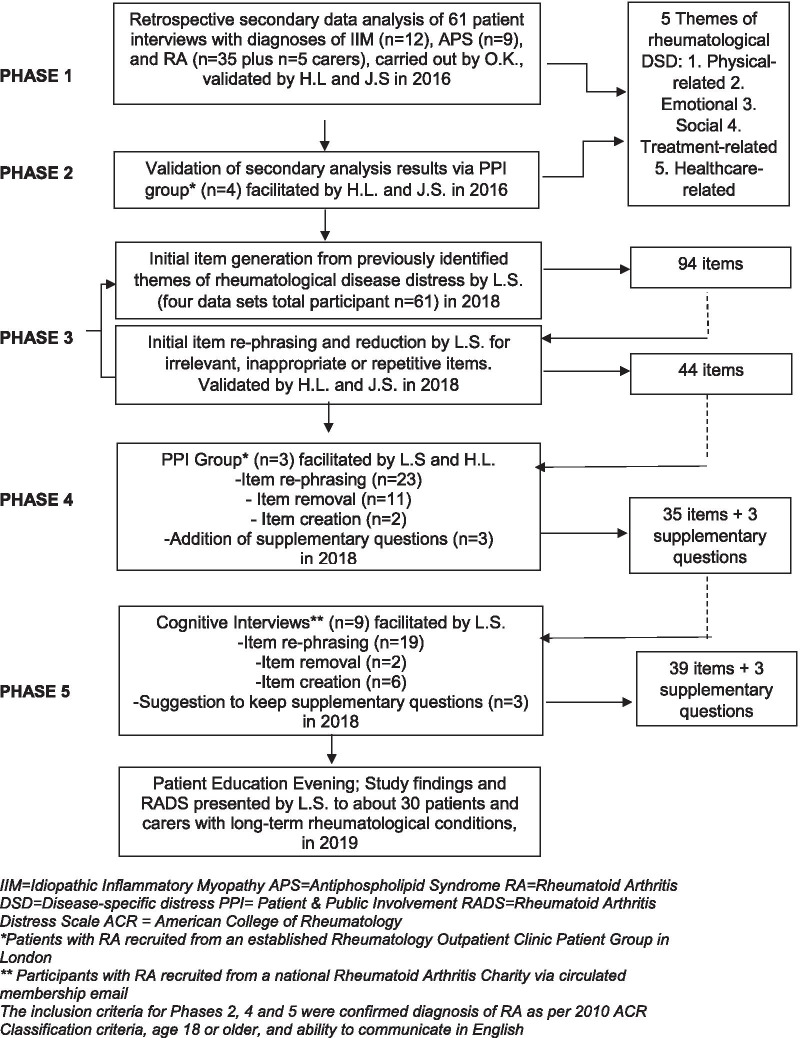
Flow chart of phases of development of the RADS

The views and perspectives of a total of 71 people with rheumatological disease plus five carers of people with rheumatological disease were involved in phases 1–5 with specific sample detail presented in Fig. 1 and Table 1.

**Table 1:** Study sample characteristics

STUDY phase/title	Data
**PHASE 1**	
Myositis fatigue study [24]	12 male and female adult patients
Anti-phospholipid Syndrome Fatigue study [25]	9 female adult patients
Rheumatoid Arthritis study [23]	26 male and female adult patients
Titrate study (intermediate Rheumatoid Arthritis) [22]	14 Adults (9 patients and 5 carers)
Total RA patients from Phase 1 original data sets	35
Total non-RA patients from Phase 1 original data sets	21
Total carers for RA patients from Phase 1 original datasets	5
**PHASE 2**	4 adults with RA
**PHASE 4**	3 adults with RA
**PHASE 5**	9 adults with RA (adult duplicate participant also from Phase 4 = 8 individual adults)
Total RA patients from Phases 1–5	50
Total non-RA patients from Phases 1–5	21
Total carers for RA patients from Phases 1–5	5

Phase 1 demographic data was previously reported [22–25] and not formally collected during Phases 2–5.

We aimed to recruit 3–5 participants for the Phase 2 and 4 PPI groups and 8–10 participants for the Phase 5 cognitive interviews, reflecting current research practice [26, 27].

### Data collection and analysis

An overview of the five phases of scale development is shown in Fig. 1.

#### Phase 1: secondary analysis

**Fig. 2:**
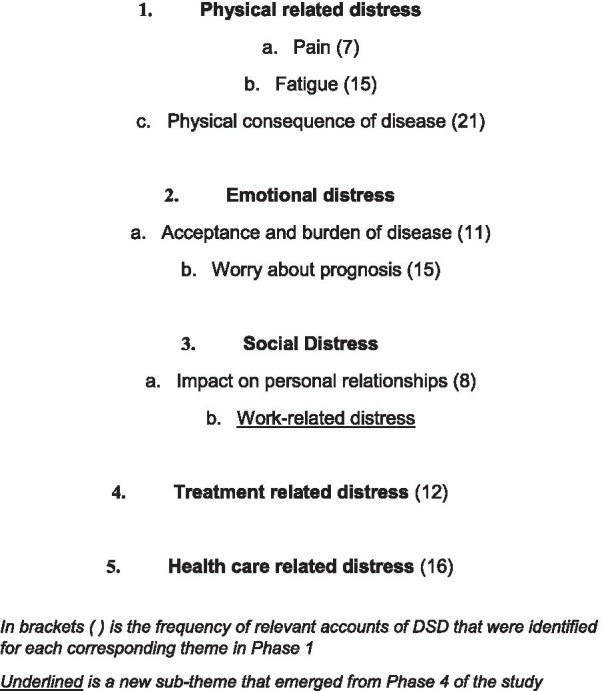
Themes of rheumatological disease distress

Using the computer software program NVivo 12, a thematic secondary qualitative data analysis was undertaken by author O.K. retrospectively from 61 audio-recorded interview transcripts from four existing data sets [22–25]. Through three codeing phases, codes were generated which identified six broad key domains: pain, fatigue, physical consequence of disease, mood, social impact and healthcare related issues. Relevant accounts from the interview transcripts were linked to the six domains, reviewed in detail and further refined looking specifically for DSD, with authors H.L. and J.S. Data that included distress was then flagged and grouped into a common ‘theme’ (see Figs. 1, 2).

#### Phase 2: validation of secondary analysis

A PPI group with patients diagnosed with RA (n = 4) was established to confirm whether the findings of the thematic analysis resonated with patients’ experiences of living with rheumatological conditions. The PPI group deliberations were audio-recorded and summarized by O.K.

#### Phase 3: initial item generation

Items for the Rheumatoid Arthritis Distress Scale (RADS) were generated from statements based on the anonymised transcripts used in the secondary data analysis, and the five themes of distress identified from that analysis (see Fig. 2). The initial list of items generated was refined by L.S. and H.L. Item order was considered at this stage by reviewing the literature [28, 29]. At the end of Phase 3, 44 items formed the first draft of the RADS.

#### Phase 4: PPI group

A unique group of patients with RA (n = 3) (who had not participated in earlier research phases) were invited and took part in a PPI group. The 44-item first draft of the RADS was presented to participants. Questions about the content, phrasing, structure, and layout of the PROM were explored. Participants were presented with alternative versions of the RADS to assess their preferences for scoring (numbers versus words). Field notes were taken from the PPI group deliberations, which were audio recorded and transcribed verbatim by L.S.

The same participants were contacted one week later via email to confirm changes made to the PROM [30]. At the end of Phase 4, a 35-item RADS was drafted with three different layout versions.

#### Phase 5: cognitive interviews

Individual face-to-face (n = 4) or telephone (n = 5) cognitive interviews were conducted with participants with RA, by L.S., to identify any difficulties in understanding PROM instructions/content or scoring. Participants were asked to ‘think aloud’ as they read each item about what they understood it to mean, and if they thought the item was relevant [31, 32]. Participants were asked to clarify how they ‘came up’ with their answer, to assess scoring discrimination [33]. These cognitive interview techniques have been described elsewhere and used in PROM development [31–33].

By Phase 5, three versions of the RADS had emerged; one scored by word phrases one by numbers and one had supplementary condition-related contextual questions about time since diagnosis and level of disease activity. The versions were alternated during cognitive interviews after every second participant, to assess for preference to score using numbers versus words.

At the end of each interview, participants were asked which version they preferred, and if they thought the supplementary questions were relevant. Field notes were taken for the interviews, which were audio recorded, and transcribed verbatim by L.S. (interviews 1–2) and professional transcriber (interviews 3–9). To avoid participant burden, these transcripts were not sent for checking [34].

Data saturation [26] was continuously assessed by L.S. through monitoring the quality and level of new information emerging from subsequent interviews.

Phase 5 transcripts were uploaded to NVivo 12. Codes were created for each item (1–35). Sub-codes within these were generated to group the qualitative data into ‘Discard’, ‘Keep’, ‘Rephrase’ and ‘Unsure’. Additional main codes created are as follows; ‘Scoring’ to analyse scoring preferences; ‘Supplementary Questions’ to analyse preferences on the additional questions; ‘Instructions Clarity’ to analyse if participants reported instructions were clear or not; ‘Instructions Time Recall’ to analyse preferences for the time recall period of the scale; ‘Concept of DSD’ to identify if participants validated the concept of DSD in RA. Data was coded by L.S. and reviewed by H.L.

Modifications to the RADS were finalised based on the data analysis of Phase 5.

### Psychometrician consultation

An academic psychometrician was consulted twice at the University to advise on the study design, scoring, numbering of items and modifications to the RADS, and recommendations for the future steps in the PROM development.

## Results

### Phase 1

Figure 2 shows the five themes of rheumatological disease distress formed from the secondary data analysis. Appendix 1 shows examples of relevant accounts from original transcripts illustrating identified themes of distress.

### Phase 2

Overall, participants in the PPI group agreed that the themes of distress resonated with their experiences of living with RA (see Appendix 2).

### Phase 3

Initial item generation yielded 94 items, categorised in order of the five themes of distress. After removal of repetitive statements, 44 items formed the first draft of the RADS. Items remained grouped in the themes of distress, but those relating to emotional distress were placed before items of physical distress to reflect the journey of patients with RA, following diagnosis, acceptance and impact of the disease. No other item order changes were made, informed by a literature review that indicated that there was no consensus for ordering of items [28, 29].

### Phase 4

Appendix 3 shows examples of relevant accounts from Phase 4 PPI group. Participants unanimously reported that the themes and concept of DSD in RA resonated with their experiences of living with the disease. An additional theme of ‘Work-related distress’ was identified, added as a sub-theme of Social Distress (see Fig. 2). A corresponding new item to this was suggested, as was a new item regarding infections.

PPI group feedback was to use word-based scoring and reduce the recall period in the PROM instructions from two months to two weeks, as a shorter duration was seen as the most reasonable amount of time one could remember accurately for scoring. Three additional supplementary questions based on time since diagnosis and disease activity were suggested, considered as contextually important for interpreting the distress score.

After the PPI group, a total of two new items had been created, 11 had been removed, and 23 had been re-phrased.

By the end of Phase 4, three versions of the RADS existed, each with the same 35 items, but differed based on word or number scoring, and the presence of supplementary questions.

### Phase 5

Thirteen members of a national RA charity expressed interest, with nine participating. Four could not take part due to scheduling conflicts.

Phase 5 interview data that was uploaded onto NVivo 12 yielded the results presented below. See Appendix 4 for relevant accounts from interview transcripts.

(i) The concept of DSD in RA

Five participants clearly validated DSD as an entity in RA and three inferred it was a valid concept when discussing certain items, for example in relation to pain.

One participant did not seem to have experienced DSD but verbalised she could see how the items were relevant and distressing for others living with RA.

(ii) Item relevancy and rephrasing

Participants (n = 9) reported that most of the items were relevant to their personal experiences living with RA or would be to others living with RA. Participants suggested minor rephrasing of 19 items.

(iii) Item creation

New items were suggested in relation to the theme of treatment-related, physical-related, and healthcare-related distress (n = 5).

(iv) Item removal or combination

Five participants reported that two items regarding RA pain were similar and could be combined, and that the word ‘distress’ better explained feelings towards their pain than ‘anger’. Two disagreed.

Seven participants reported that two items concerning test results and healthcare professionals’ explanations of treatments were similar and could be combined.

(xxii) Item order

Participants had few strong opinions on the order of items on the PROM and stated that the current sequence made sense. However, six suggested that item ‘I feel overwhelmed living with RA’ could move to the end of the scale.

(vi) Supplementary questions

Nine, seven and six participants preferred to keep supplementary questions 1, 2 and 3 in the scale, respectively. Participants reported that the questions gave context to scoring the PROM. Data regarding participants’ specific answers for supplementary questions and disease activity scoring was not collected.

(vii) PROM scoring

Six participants reported preferring to answer the PROM using word scoring, two preferred numbers, and one held no preference.

Participants who preferred word labels reported being able to ‘relate’ more to word scoring and helped them to consider each item more carefully than if scoring on a numerical scale.

(viii) PROM instructions

Overall (n = 9), participants reported that they understood PROM instructions and they were clear. Regarding the timeframe mentioned in the instructions, they reported that due to the variability of their condition, two weeks may not accurately capture distress levels.

Some participants (n = 3) suggested to extend the timeframe to one month, some (n = 1) to three months, some (n = 2) were unsure, while others (n = 3) agreed with two weeks.

### Demographic data

Although demographic data was not formally collected for Phases 2, 4 and 5 participants, some information unintentionally was disclosed; Most participants were female, Caucasian, and revealed their occupations were in the banking, business, or healthcare-sectors. Disease duration varied from two to 40 years in Phase 5 participants.

### Psychometrician consultation

The psychometrician validated the study design and confirmed word-based scoring to be appropriate for a PROM so long as presented in ordinal format for summation of scoring. The psychometrician also confirmed the supplementary questions as relevant and suggested these to be inserted at the end of the PROM to avoid potentially influencing participants scoring. Finally, the psychometrician suggested consideration of the time recall period in the instructions to ensure consistency and validity of participant answers.

### Final changes to the RADS

Final changes were made to the RADS based on the results and analysis from Phase 5 data presented above (See Table 2 for final item list and RADS Final Version in Appendix 5).

**Table 2:** Rheumatoid Arthritis Distress Scale (RADS) items and response options

Items
1. I find it difficult to accept having RA
2. I feel that having RA has a big impact on me
3. I feel worried that having RA has a big impact on my family or friends
4. I feel worried that I might have to depend on family or friends in the future
5. I find it difficult to accept the impact my RA might have on my ability to work
6. I am concerned that my disease might not be well controlled
7. I worry about the long-term impact of my disease
8. I worry about having other long-term conditions in addition to my RA
9. I am worried about the impact of developing infections due to my low immunity
10. I am concerned that medication will not stop the disease progression (including joint damage)
11. I feel frustrated that there is no cure for RA
12. I feel distressed because of my RA pain
13. I feel frustrated because my RA symptoms limit my mobility
14. I feel irritated because my RA symptoms disrupt my sleep
15. I feel frustrated because of my fatigue associated with my disease
16. I feel frustrated because sleep does not relieve the fatigue I feel with my disease
17. I feel frustrated that my RA stops me doing what I want to do
18. I feel my energy is drained living with RA
19. I feel frustrated that I cannot do everything I used to be able to do/enjoy
20. I feel frustrated that I cannot do everything I would like to be able to do
21. I feel frustrated that I cannot be as physically active as other people my age
22. I feel distressed trying to manage my weight with having RA
23. I find it frustrating that people do not understand RA
24. I am concerned my RA will have an impact on my ability to look after others
25. I am frustrated that I do not have enough support to enable me to do a/my job
26. I feel a loss of purpose because I cannot work/work to the extent I used to due to my RA
27. I worry that having RA may affect my finances
28. I feel deflated about different RA treatments not working effectively for me
29. I am frustrated about the side effects of my treatment
30. I feel distressed with the regimen of collecting and managing my medication
31. I feel frustrated with the difficulty in accessing help from healthcare professionals e.g. accessing nurse help line
32. I am frustrated when clinic appointments are cancelled or rescheduled at short notice
33. I feel frustrated at the lack of continuity of my care e.g. seeing several different consultants
34. I worry that attending so many appointments for my RA impacts on my other commitments
35.I feel frustrated when healthcare professionals do not take enough time to assess my condition
36. I feel frustrated when healthcare professionals do not adequately explain test results or treatments to me
37. I feel frustrated that healthcare professionals do not ask how I am coping living with RA
38. I feel frustrated that I am not adequately supported or listened to by healthcare professionals
39. I feel overwhelmed living with RA

When thinking about the level of distress living with RA may cause, how serious a problem is it? Response options are “Not a problem”, “Slight problem”, “Moderate problem”, Serious Problem” or “Very serious problem”

A preliminary decision was made, following PPI -group discussions, for instructions to score based on the previous three months rather than two weeks, ensuring enough timeframe to capture different aspects of distress. Word-based scoring was chosen as the predominant preference from Phase 4 and 5 participants. No major differences were noted from gross observation of scoring patterns when alternating the different versions in Phase 5.

In total, 19 items on the PROM were rephrased, two were removed, and six new items were created. The supplementary questions were retained in response to the participants’ preference, and the psychometrician’s advice.

For item order, new items were inserted into the PROM alongside those from their corresponding theme. Item ‘I feel overwhelmed living with RA’ was moved to the end to become item 39 on the RADS as per the participants’ (n = 6) suggestions. Therefore, after analysis of the data in Phase 5 of the RADS development, the final version consisted of a 39-item scale with three supplementary questions (See Appendix 5).

## Discussion

### Presentation of principle findings

This study described the five initial phases of development of the RADS, a PROM to identify DSD in people with RA. The PPI group and cognitive interviews confirmed empirically the findings from the secondary data analysis that RA distress does exist as an important entity and appears distinct from other conditions like clinical depression. The 39-item RADS demonstrates initial face and content validity with people with RA. Conceptually, the 39 items link to one of five themes of RA distress. The RADS is now available for further psychometric evaluation in clinical and research populations.

### Comparisons of evidence with the wider literature

To the authors’ knowledge, this is the first PROM developed to identify DSD in people with RA. The Rheumatoid Arthritis Impact of Disease (RAID) questionnaire measures seven domains of disease impact [35]. However, the RADS focuses in more detail on the emotional distress and cognitive impact that is commonly experienced in RA, specifically on the symptoms, burden and treatment.

The themes of RA distress (Fig. 2) from this study are similar to those identified in type 1 and 2 diabetes [19, 36], IBD [9] and to a lesser extent cancer distress scales [37]. These long-term diseases share constructs of emotional, healthcare-related, treatment-related, and social distress. Emotional distress items from two validated diabetes distress measures [19, 36] echo the RADS, describing difficulties of illness acceptance and worries about future complications. In this study the predominant emotion for people with RA seemed to be of frustration, although minor themes of anger also emerged, with some participants reporting that anger was not an emotion they were ‘allowed’ to feel or express, resonating findings from previous qualitative studies [38]. In diabetes emotional health PROMs, distinctions are drawn between appraisal of emotional impacts such as distress and assessment of quality of life [39]. In assessing distress people talk about feelings such as worry and frustration. In cognitive assessments people use terms such as “I think” or “being concerned about” something. Three of the 39 items [6, 10, 24] use cognitive appraisal terms with the remainder using emotional phrases consistent with the diabetes distress PROMs [19]. Further item redundancy may be identified in future psychometric evaluation.

Cancer [37], diabetes [19, 36], IBD [18] and RA scales have all included items about healthcare-related distress. In diabetes and IBD ‘concerns not taken seriously’ by healthcare professionals emerged, while in RA lack of clinicians’ time spent to assess the condition and provide emotional support caused distress. In both cancer and RA, distress over lack of adequate information from healthcare professionals has been described [37].

Treatment-related distress in IBD and RA included concerns over side effects, while in diabetes lack of confidence/motivation in self-management, and guilt about failing with treatment regimens, seems predominant. Social-related distress common to all four long-term conditions highlighted a lack of understanding from others about their illnesses.

People with RA, IBD or cancer, but not diabetes, demonstrate symptom-related distress. People with RA are distressed about their joint pain, stiffness, and fatigue. Pain may explain the difference in degree of depression between RA and healthy controls [40] and there is some evidence that psychological distress in RA can be secondary to pain rather than vice versa [41]. The link between pain and DSD in RA, however, has not been previously investigated.

Elevated diabetes distress is prevalent in 20–40% of people with type 1 [42, 43] and in 36% of people with type 2 [44] diabetes. Four systematic reviews have explored psychological interventions in diabetes. In general, reviews found psycho-educational treatments resulted in a low to moderate effect on DSD reduction and more intensive and longer duration interventions seem to achieve a greater effect [13, 14, 45, 46]. One systematic review demonstrated that motivational interviewing significantly reduced diabetes distress and improved glycaemic control [13]. Furthermore, diabetes-tailored interventions, as opposed to general mindfulness interventions, showed to most likely improve both DSD and glycaemic control in people with type 1 and 2 diabetes [14].

In a cross-sectional study of 189 cancer patients, 58% demonstrated elevated DSD [6]. Psychosocial interventions, such as cognitive behavioural therapy and meaning-centred psychotherapy may improve quality of life and alleviate anxiety in people with cancer, although systematic reviews have not focussed on DSD as a primary outcome [47, 48].

In IBD and RA, the prevalence of DSD is not yet known, nor what effective treatment may entail. However, psychological interventions such as supportive counselling can result in small to moderate improvements in biopsychosocial outcomes for patients with RA [49].

### Strengths and limitations of the study

The strengths of this research are its systematic study design, which was validated by a psychometrician, and followed recommended guidelines of PROM development [20] and reporting [21]. The secondary data analysis was an ethically efficient way to make good use of anonymised data. People with RA were involved throughout the process of development. Changes to the PROM after Phase 4 were endorsed by PPI participants, ensuring accurate interpretation of data [30]. The robustness and validity of data was further strengthened by inclusion of diverse cases/minor themes [21]. One notable strength of our demographics was the mixture of early and established RA.

The PPI groups in this study had taken part in several similar research projects in the past and embraced the topic of RA distress. The data generated therefore led to high ‘information power’ [50]. The final two cognitive interviews in Phase 5 did not yield as much ‘new information’ indicating close ‘data saturation’ [26].

This research has limitations. Our study would have benefited from a larger sample size for the PPI groups and cognitive interviews, including more males and people from diverse socioeconomic and ethnic backgrounds. RA incidence can be 4–5 times higher in females [51], hence challenges in recruiting males for this study. Cognitive interview participants were recruited from one national charity, which could explain the homogeneity of this sample. The team did not send participants transcripts for fact/content checking to minimise research burden and/or fatigue [34].

We included data in our analysis from participants who were carers (n = 5) to people with RA and diagnosed with APS (n = 9) and IIM (n = 12), in addition to people with RA (n = 50). Therefore, it is possible our data captured evidence of rheumatological disease distress rather than isolated RA distress. As 60% of our participants had a confirmed diagnosis of RA, the authors concluded to present this PROM as an RA distress scale, while acknowledging that there is likely overlap in distress measures across rheumatic diseases. We cannot exclude similar overlap in causes of distress amongst patients who may have un-diagnosed co-morbid Fibromyalgia or central sensitisation disorders.

Finally, participants in Phase 1 were not specifically asked about RA distress and since the 39 items were generated during secondary analysis of this data, some items areas may be missing from the RADS.

### Recommendations for practice, policy and research

DSD in RA appears to be a new important concept and its identification comes at a pivotal time; the 2018 updated NICE guidelines for RA management in adults recommended clinicians to assess the effect RA has on the patients’ life [52]. Furthermore, patients with RA report that the most frequent reasons for flare up of joint symptoms is psychological stress [53]. It therefore is important to identify this subset of psychological distress.

With the development of the RADS it is hoped that DSD can be identified in RA to build on interventions to reduce distress, as has been achieved in type 1 and 2 diabetes [13], whereas the RAID appears to be useful more in monitoring the impact of RA on patients’ lives.

There is a need to establish face and content validity of the RADS in a more diverse patient population. Cut-off thresholds for severity need to be explored as currently the measure does not have a scoring range or item weighting. Item order could be investigated for reliability differences between item grouping or intermixing. Future studies need to focus on test–retest reliability, cross-sectional and longitudinal construct validity. Feasibility needs to be evaluated, including scoring time; The RADS is long with 39 items to record. Further psychometric evaluation will clarify additional potential item redundancy.

Currently no gold standard for RA distress exists. Therefore, the RADS could be evaluated against modified validated diabetes and/or IBD distress scales, considering the similarities in distress domains.

Following detailed psychometric evaluation, the RADS has the potential to be employed in larger longitudinal studies to identify the prevalence of RA distress. It would be worth investigating whether DSD is associated with clinical outcomes in RA. Effective psychological interventions for DSD, as implemented in type 1 and 2 diabetes patients [13], would be welcome by people with RA and their carers.

## Conclusions

In summary, this study offers evidence for DSD as an important entity experienced by people with RA. It appears RA distress shares some domains with other long-term condition specific anguish. The 39-item RADS has acceptable first phase face and content validity in people with rheumatological long-term conditions specifically RA. RA distress appears to be distinct from clinical depression or anxiety disorders. The RADS has the potential to be a useful tool for identifying RA distress.

## Data Availability

The datasets used and/or analysed during the current study are available from the corresponding author on reasonable request.

## Appendix 1: Accounts from original data sets used in Phase 1

The below accounts are some examples from the original data sets used in Phase 1 secondary analysis that illustrate the five themes of rheumatological disease distress.

- **1. Physical related distress**

- **1a. Pain**

- **Distress related to the physical symptoms of RA such as pain was commonly identified. There was a range of negative emotions expressed within the interviews including low mood, frustration, fear and anger as well a sense of helplessness and feeling overwhelmed with the long-term condtion.**

I was annoyed. I was really annoyed with myself. It’s like, I’m getting up to do something, and you can’t move. I lived on the settee for about 6 or 7 months; I couldn’t get up the stairs. I couldn’t sleep through pain. **(Patient 12, RA).** You’re just in pain and you’re crying. You couldn’t do anything, so you just lay there suffering, basically. That’s what you did, is suffer. And that was horrible. **(Patient 13, RA).**

**1b. Fatigue**:

- **RA and other autoimmune conditions are systemic conditions that can cause fatigue. It was a common finding amongst patients and had a significant impact on their quality of life.**

The fatigue it frustrates me. That’s one of my main frustrations. I’ve come to terms with the disease and I know I have got to change my lifestyle, but it still frustrates me sometimes that I can’t do what I want to. **(Patient 4, IIM).** But sometimes you’ve just got to succumb to how the body’s feeling. Yeah, you can’t fight it. It’s a tiredness that overwhelms you, I think. **(Patient 21, RA).**

- **1c. Physical consequence of disease**

- **Distress related to the fear and loss of function as well as disability was re-occurring themes across all patients that were interviewed across all studies**.

I feel angry, I like to be independent, I like to do things for myself and I like to be myself. I just don’t like to be dependent. I feel angry if I can’t do anything. **(Patient 19, RA).** It worries me, it worries me for practical things, like when my disability allowance comes up for renewal, who the hell is going to fill the form that end and say what I do and don’t need, because they have no idea. They have no idea the level of my disability on day to day activities. **(Patient 1, IIM).** I am very worried if it [RA] continues like this I am worried that I may be paralyzed one day. If it helps this intensive introduction of medicines then I will work on it. Because the doctor told me himself that the percentage that I will one day be paralysed is very high. And I am very horrified. **(Patient 10, RA).**

- **2. Emotional distress**

- **Emotional distress was also commonly identified; negative emotions of hopelessness, frustration and anger were often expressed. Feelings of being overwhelmed by living with the physical demands of their long-term condition (e.g. accepting help for activities of daily living, attending appointments, taking medication) were often reported.**

- **2a. Acceptance and Burden of disease**:

- **Some patients were not accepting their diagnosis, which caused emotional distress**:

… it was hard, it is hard more or less accepting it. It was so depressing and all that. There were times when you… just wanted to give up, but looking at the kids, I say: ‘no I got to hang on’. I must do it. They [doctors] said the medicine is going to work, so I should hang on in there. They will work. **(Patient 7, RA).**

- **Some patients were overwhelmed by the burden of their disease and distressed due to their dependence on others**:

I do tend to get very depressed at times. …I can’t go on holiday unless someone in the family takes me on holiday. I have to rely on people so much. I just get so wound up with myself. **(Patient 1, IIM).** I wouldn’t say I’m depressed. Like clinically depressed with the correct use of the term….so many people say, ‘oh I’m stressed, I’m depressed’, but I would say, yeah I just get fed up that I can’t do what I want to do really, although I do still do a lot, even though I’m not particularly one hundred percent there. **(Patient 13, APS).**

- **Distress related to how the burden of disease can impact and restrict the patients as well as their partner’s daily activities:**

I hate being a burden. Although they’re very good, all of them [family] and especially my husband. But I don’t want to be a burden, you know. **(Patient 14, RA).** It all sort of stops me doing what I want to do sometimes. You know, sometimes people just think that you’re lazy. It’s not that I don’t want to do it, I just can’t. **(Patient 11, APS).**

- **2b. Worry about prognosis**

- **Patients were frequently overwhemled with what the future may hold in terms of their overall disease prognosis and fear of further progression**.

It’s hard sometimes, because I’m scared to go to bed, because I’m going to wake up in the morning and I don’t know what will happen. **(Patient 22, RA).** The biggest stress for me is what I have been told by the bone specialist, every time I am going. I was told this is the kind of diseases that will continue, I will continue having this (RA) and it’s not going to disappear. It is a very stressful thing. **(Patient 9, RA).** How does it feel that you can’t rely on your body really? I’m worried about the future because, like, I don’t feel my age. **(Patient 8, RA).** I’ve no backup of scientific evidence but I also believe that the more things that happen to my body, your body has to work as a whole, and there are all these things that after that time of years of age that happen to my body, is going to have an effect on the way I function as a person. **(Patient 11, APS).**

- **3. Social Distress**

- **3a. Impact on Personal Relationships**

- **Patients with long-term rheumatological conditions were worried about how their disease would impact on their family life/dynamics. Feelings of helplessness were expressed e.g. with not being able to provide due to their disease.**

Because my daughter is pregnant and also I have two small grandchildren and I am worried for them, that’s why I am scared. My daughter is living separately but my grandchildren are living with me. I am concerned for them as well. **(Patient 1, APS).** I know if I was on my own, life would be very, very difficult. I mean, I’ve got family and everything, but they don’t want to live with you, they don’t want you living with them. **(Patient 10, RA).**

- **A lack of appreciation of the difficulty of living with RA, as well as the lack of emotional support from friends and people around them was also found in the data**:

You’re fighting with how much that illness has changed your life. Sometimes people, because you’re in a chronic situation, sometimes friends might find it difficult to deal with (illness), because they don’t know…Because in a way, it’s like a form of bereavement. **(Patient 21, RA).**

- **3b Work-related distress****

- **The data suggested that distress from dissatisfaction with employers largely based on lack of empathy and understanding of their illness and physical limitations**

They [employers] didn’t understand my disease and the fact that they could have helped by keeping my workload at a certain level. And however much I told them that if I had to do more, it would make me iller, I just don’t think they could understand. **(Patient 24, RA)**

- **4. Treatment related distress**

- **Distress generated by the prospect of taking regular, daily lifelong medication was also found**:

I don’t like being dependent on other people. And the other thing I don’t like is taking medication. So, you know, I hate… Until this illness I don’t think I’ve taken a pill in my life! **(Patient 9, RA).**

- **Side effects of medication also caused distress**:

Think actually, next time I see Dr XX, I’ll ask him to sort out…because I’m taking these Methotrexate which don’t make me feel good, they don’t seem to do anything! **(Patient 15, RA).** I couldn’t cope with. It was awful! And I was really cross with myself that I couldn’t get to grips with it. **(Patient 2, RA).**

- **Lack of efficacy from medication**:

The tablets are not working and the doctor can do nothing more. You see there can’t do nothing more, because that [medication] has gone on for a long time. **(Patient 4, RA).**

- **5. Health-care related distress**

- **Distress also related to concerns expressed by patients that they were not taken seriously by their health care professionals.**

No, he (clinician) is not interested in either of that (how I manage at home). He is very much interested in the drug regime, the medical bit. And also since they got the new computer system, how he manages to put it [data] on the screen and that sort of thing. He is obsessed about, how do I put this on this file, he spends more time talking to the computer than he does to you. **(Patient 1, RA).** My life is just taken over by going to the doctor's—going to the hospital for check-ups or whatever. I've just joined myself onto a volunteer thing to do voluntary work, and I'm a bit worried now, because I have to go to the hospital so many times that I don't know if I'm going to be able to do it. **(Patient 4, RA – Titrate).** I’m still on the same medication and, you know, he hasn’t looked at me; he hasn’t examined me; he hasn’t said to me: “We’re going to change your medication.” I mean, the last time I went there he said to me:”How do you feel?” I said: “My joints are hurting me more,” and all that. So he said to me: “Alright, we’ll increase your Methotrexate.” So he put it up from 8 tablets up to 9 tablets. **(Patient 3, RA).** He does not really understand, he’s never heard of it and he was the one that said that it was arthritis so… I actually ended up, before I got diagnosed, ended up having an arthroscopy and the surgeon that did it said ‘you don’t have arthritis at all’ and that’s when I got referred to Dr… **(Patient 5, IIM).**

- **Lack of communication between health care professionals and patients created further distress and anxiety, e.g. not having blood test results explained**

I don’t know the results, I never; I like to know the results, because I don’t know about the results. They just take blood, and that so annoys me and I don’t know the results. I want to know. **(Patient 19, RA).** ** Although sub-theme of Work-Related Distress did not emerge until the Phase 4 Patient and Public Involvement (PPI) group in this study, as can be seen from these transcripts, distress surrounding work issues can be seen from Phase 1 data.

## Appendix 2: Accounts from Phase 2 PPI group

- **1. Physical related distress**

- **a. Pain/disability**:

Patients agreed that pain from their RA was physically overwhelming at times and especially distressing, impacting on all aspects of their daily living resulting in disability. You’re in so much pain you really cannot do what you want to do (Patient 1)

- **b. Fatigue**

Patients unanimously agreed that fatigue was one the largest physical stressors, affecting jobs, relationships and daily living. This persistent and debilitating symptom generated much distress. Fatigue is a killer. I want to do more, but you can’t do it, it seems to come over you. (Patient 4)

- **c. Physical consequence of disease**

Examples of distress relating to loss of function and disability emerged although focus group time limitations meant this sub-theme was not explored in depth. ..not being able to do things, I just cannot move. It’s really frustrating and people can’t understand it (Patient 4) You’re unable to rely on your body which is really frustrating (Patient 1)

- **2. Emotional and social distress**

- **2a. Acceptance and burden of disease:**

Some of the patients were still finding it hard to accept the diagnosis of RA, whilst others were stressed from the physical demands of their disease (i.e. feeling as though they are a burden on relatives). I have had RA 5 years, I am yet to accept it, it’s a constant battle (Patient 3)

- **2b. Worry of prognosis**

Some patients were distressed about progression of their disease, affecting their ability to work and keep their job to provide for their family. Others were also concerned about the outlook and uncertainty and the course that their disease will run (i.e. leading to disability). Fluctuation and uncertainty…(can be distressing) (Patient 1)

- **3. Social distress**

- **3a. Impact on personal relationships**

The distress on relationships was emphasized with patients expressing views about the lack of understanding from society (i.e. employers, public) about their disease, and the lack of acceptance and empathy. The stress of being a burden to their relative from their physical disease was also found. I think it’s very hard on partners, and a strain on the relationship, they just get fed up with you (Patient 3)

- **4. Treatment related distress**

The patients confirmed that there was much anxiety and worry about taking their medication for their illness. Patients were distressed about compliance due to side effects, lack of efficacy and disease progression. I’m worried about my liver with the medication (Patient 2)

- **5. Health care related distress**

Patients identified that they were often distressed about the frequency of outpatient appointments that they had to attend. Others were frustrated by their clinicians lack of emotional understanding especially on how their disease is impacting their daily life (physically and mentally). I had a bad experience in the orthopedic clinic. He put the X-ray of my hand up...he said I haven’t seen hands like this for a long time…they’re awful aren’t they… (Patient 3)

## Appendix 3: Accounts from Phase 4 PPI Group

- **On the concept of DSD in RA:**:

I’m a firm believer in DSD. It was like a switch [when I heard about it]. Because I’m not clinically depressed. But when you fill out some forms [in clinic] I know what I was putting made it look like I did. What I had answered I came out being clinically depressed because I had a bad week.” (Person A)

- **On new sub-theme of Work-Related Distress, and new item suggested based on this**:

One thing is missing for me. I do get anxiety and I do get low but it’s not clinical depression. It’s a lot to do with giving up work, and that had a big impact on me. So I think work is missing there [in the scale]. (Person A) I would put it [the new theme] under social distress. For me, it’s complete loss of identity. Because I’m not married, once that role was taken away from me, I had to leave my job, my flat in XXX. I’d gone from managing people in a large company to sitting at home in my flat in XXX. It takes getting used to. You lose your sense of purpose. (Person B)

- **On new item regarding distress around infections**:

I think that’s very relevant. We all know we’re immunosuppressed. I am very aware if I’m on the tube and someone’s sneezing or coughing, I am holding my breath! (Person A) So I’d put in a question ‘I’m worried about catching a infection’ and put in brackets ‘due to low immunity’ (Person B)

## Appendix 4: Accounts from Phase 5 cognitive interviews

(ix) *The Concept of DSD in RA*

Five participants clearly validated DSD as an entity in RA, for example: I go through periods of being particularly, I would say suffering from DSD, and really get annoyed when doctors say, “Oh you’re suffering from depression,” because I think I’m not, this is something completely different. (Patient 3)

Three participants inferred that it was a valid concept, when discussing certain items, for example in relation to pain: The pain does cause distress, not because of the physical pain itself because with time most people learn to live with it, but distress is from the implications of the pain, what’s causing the pain…there’s a whole lot of things you can’t do. That’s what’s distressing not the pain itself. (Patient 1)

(xxiv) *Item Relevancy and Rephrasing*

Participants suggested minor rephrasing of 19 items. For example, seven participants reported that regarding item 1, finding it difficult to accept *having* RA would be more relevant than accepting being *diagnosed* with RA, which they thought would only be applicable to people who were recently diagnosed: I think having RA would be much more relevant because that impacts you more… I think maybe I accepted being diagnosed straightaway, I don’t know whether I accepted having RA. (Patient 8)

(xi) *Item Creation*

New items were suggested in relation to the domain of treatment-related distress, I feel distressed with the regimen of collecting and managing my medication (n = 1) physical-related distress, I am frustrated that I cannot do everything I would like to be able to do because of my RA (n = 1) I feel distressed trying to manage my weight with having RA (n = 1) and healthcare-related distress: I feel frustrated at the lack of continuity of care (n = 1) I feel frustrated that I am not adequately supported by the healthcare system (n = 1) I feel frustrated that my care is not co-ordinated (n = 1) I feel frustrated with the difficulty in accessing help from healthcare professionals (n = 1) I am frustrated when clinic appointments are cancelled or rescheduled at short notice (n = 1) Most of the new items suggested (n = 5) were based around healthcare-related distress, which for one participant was the main source of frustration: This is one of the issues I have with my treatment for my RA over the years, is that talking about the distress side of things, a lot of the distress has been caused by standards and the way I’ve been treated. (Patient 7)

(xii) *Item Removal or Combination*

Five participants reported that items 12 and 13 were similar and could be combined, and that the word ‘distress’ better explained feelings towards their RA pain than ‘anger’: Distress would be a...[better word]. I didn’t feel angry at all because of the pain. It’s just not an emotion that I generally feel so I wouldn’t identify with that. (Patient 8) Two participants, however, felt anger was an appropriate emotion for their pain. One participant remarked: It’s not something we’re allowed to say often…you’re just supposed to cope with it and deal with it and that’s it. So I quite like angry. (Patient 4)

(xiii) *Item Order*

Six participants suggested that item 20 could go at the end of the Scale: Put question 20 at the end as it’s sort of a summary question ‘being overwhelmed’. (Patient 2)

(xiv) *Supplementary Questions*

Participants reported that the questions gave context to scoring the scale: The first [supplementary] question is about when your diagnosis was. Because I think I’d answer this differently two years ago when I was first diagnosed. (Patient 2)

(xv) *Scale Scoring*

Participants who preferred word labels based their decision on being able to ‘relate’ more to words and reporting that it helped them consider each item more carefully than if scoring on a numerical scale: I always just find I’m guessing a number whereas I’d prefer to give a word to describe it, but that’s obviously a personal interpretation. (Patient 4)

(xvi) *Scale Instructions*

Regarding the scale instructions timeframe, participants reported that due to the variability of their condition, two weeks may not accurately capture distress levels: The only thing that flags for me is the past two weeks could have been amazing and the two weeks before that could have been hell, and for me the past two weeks haven’t been that bad. (Patient 4)

## Appendix 5: Final version of the RADS

- **Rheumatoid Arthritis Distress Scale (RADS)**

Living with Rheumatoid Arthritis (RA) can be challenging and may cause distress. This distress can arise from difficulties faced when living with RA, for example the symptoms you experience, or the impact having RA has on your relationships.

Listed below are 39 different items that may cause distress (e.g. frustration, worry, concern or anger) in people living with RA. Please read each item below and think about the level of **distress** it may be causing you. Please score the extent to which each item has been a problem for you **DURING THE PAST 3 MONTHS** by circling one answer in each line for each statement that is most appropriate to you. For example, if a particular item has not been an issue for you please circle ‘Not a problem’ whereas if it has been very distressing you might circle ‘Very serious problem’. If a statement does not apply to you, please circle ‘Not a problem’.

**Table:**

	When thinking about the level of distress this may cause, how serious a problem is it?				
1. I find it difficult to accept having RA	Not a problem	Slight problem	Moderate problem	Serious problem	Very serious problem
2. I feel that having RA has a big impact on me	Not a problem	Slight problem	Moderate problem	Serious problem	Very serious problem
3. I feel worried that having RA has a big impact on my family or friends	Not a problem	Slight problem	Moderate problem	Serious problem	Very serious problem
4. I feel worried that I might have to depend on family or friends in the future	Not a problem	Slight problem	Moderate problem	Serious problem	Very serious problem
5. I find it difficult to accept the impact my RA might have on my ability to work	Not a problem	Slight problem	Moderate problem	Serious problem	Very serious problem
6. I am concerned that my disease might not be well controlled	Not a problem	Slight problem	Moderate problem	Serious problem	Very serious problem
7. I worry about the long-term impact of my disease	Not a problem	Slight problem	Moderate problem	Serious problem	Very serious problem
8. I worry about having other long-term conditions in addition to my RA	Not a problem	Slight problem	Moderate problem	Serious problem	Very serious problem
9. I am worried about the impact of developing infections due to my low immunity	Not a problem	Slight problem	Moderate problem	Serious problem	Very serious problem
10. I am concerned that medication will not stop the disease progression (including joint damage)	Not a problem	Slight problem	Moderate problem	Serious problem	Very serious problem
11. I feel frustrated that there is no cure for RA	Not a problem	Slight problem	Moderate problem	Serious problem	Very serious problem
12. I feel distressed because of my RA pain	Not a problem	Slight problem	Moderate problem	Serious problem	Very serious problem
13. I feel frustrated because my RA symptoms limit my mobility	Not a problem	Slight problem	Moderate problem	Serious problem	Very serious problem
14. I feel irritated because my RA symptoms disrupt my sleep	Not a problem	Slight problem	Moderate problem	Serious problem	Very serious problem
15. I feel frustrated because of my fatigue associated with my disease	Not a problem	Slight problem	Moderate problem	Serious problem	Very serious problem
16. I feel frustrated because sleep does not relieve the fatigue I feel with my disease	Not a problem	Slight problem	Moderate problem	Serious problem	Very serious problem
17. I feel frustrated that my RA stops me doing what I want to do	Not a problem	Slight problem	Moderate problem	Serious problem	Very serious problem
18. I feel my energy is drained living with RA	Not a problem	Slight problem	Moderate problem	Serious problem	Very serious problem
19. I feel frustrated that I cannot do everything I used to be able to do/enjoy	Not a problem	Slight problem	Moderate problem	Serious problem	Very serious problem
20. I feel frustrated that I cannot do everything I would like to be able to do	Not a problem	Slight problem	Moderate problem	Serious problem	Very serious problem
21. I feel frustrated that I cannot be as physically active as other people my age	Not a problem	Slight problem	Moderate problem	Serious problem	Very serious problem
22. I feel distressed trying to manage my weight with having RA	Not a problem	Slight problem	Moderate problem	Serious problem	Very serious problem
23. I find it frustrating that people do not understand RA	Not a problem	Slight problem	Moderate problem	Serious problem	Very serious problem
24. I am concerned my RA will have an impact on my ability to look after others	Not a problem	Slight problem	Moderate problem	Serious problem	Very serious problem
25. I am frustrated that I do not have enough support to enable me to do a/my job	Not a problem	Slight problem	Moderate problem	Serious problem	Very serious problem
26. I feel a loss of purpose because I cannot work/work to the extent I used to due to my RA	Not a problem	Slight problem	Moderate problem	Serious problem	Very serious problem
27. I worry that having RA may affect my finances	Not a problem	Slight problem	Moderate problem	Serious problem	Very serious problem
28. I feel deflated about different RA treatments not working effectively for me	Not a problem	Slight problem	Moderate problem	Serious problem	Very serious problem
29. I am frustrated about the side effects of my treatment	Not a problem	Slight problem	Moderate problem	Serious problem	Very serious problem
30. I feel distressed with the regimen of collecting and managing my medication	Not a problem	Slight problem	Moderate problem	Serious problem	Very serious problem
31. I feel frustrated with the difficulty in accessing help from healthcare professionals e.g. accessing nurse help line	Not a problem	Slight problem	Moderate problem	Serious problem	Very serious problem
32. I am frustrated when clinic appointments are cancelled or rescheduled at short notice	Not a problem	Slight problem	Moderate problem	Serious problem	Very serious problem
33. I feel frustrated at the lack of continuity of my care e.g. seeing several different consultants	Not a problem	Slight problem	Moderate problem	Serious problem	Very serious problem
34. I worry that attending so many appointments for my RA impacts on my other commitments	Not a problem	Slight problem	Moderate problem	Serious problem	Very serious problem
35. I feel frustrated when healthcare professionals do not take enough time to assess my condition	Not a problem	Slight problem	Moderate problem	Serious problem	Very serious problem
36. I feel frustrated when healthcare professionals do not adequately explain test results or treatments to me	Not a problem	Slight problem	Moderate problem	Serious problem	Very serious problem
37. I feel frustrated that healthcare professionals do not ask how I am coping living with RA	Not a problem	Slight problem	Moderate problem	Serious problem	Very serious problem
38. I feel frustrated that I am not adequately supported or listened to by healthcare professionals	Not a problem	Slight problem	Moderate problem	Serious problem	Very serious problem
39. I feel overwhelmed living with RA	Not a problem	Slight problem	Moderate problem	Serious problem	Very serious problem

- Please indicate (in years and months) the time since your initial RA diagnosis:
  ______________________________________
- Has your Rheumatology team advised you that your RA is currently…*(please circle one answer or leave blank if unknown)*

**Table:**

In remission	Slightly active	Moderately active	Severely active

- What stage do *you* feel your RA is in currently? *(please circle one answer or leave blank if unsure)*

**Table:**

In remission	Slightly active	Moderately active	Severely active

## Abbreviations

RA: Rheumatoid Arthritis
DSD: Disease-specific distress
IBD: Inflammatory Bowel Disease
PROM: Patient Reported Outcome Measure
APS: Antiphospholipid Syndrome
IIM: Idiopathic inflammatory myopathy
PPI: Patient and Public Involvement
RADS: Rheumatoid Arthritis Distress Scale
RAID: Rheumatoid Arthritis Impact Disease
